# Pathological Predictors of Limited Salvage Radiotherapy Efficacy After Radical Prostatectomy: Central Review of JCOG0401

**DOI:** 10.3390/cancers18121868

**Published:** 2026-06-08

**Authors:** Masashi Kato, Toyonori Tsuzuki, Akira Yokomizo, Takahiro Kimura, Keita Sasaki, Masaki Shiota, Keiichiro Mori, Takuma Kato, Takashi Kawahara, Dai Koguchi, Katsuyoshi Hashine, Mikio Sugimoto, Masatoshi Eto, Hiroyuki Nishiyama, Hiroshi Kitamura

**Affiliations:** 1 Department of Urology, Japanese Red Cross Aichi Medical Center Nagoya Daiichi Hospital, 3-35 Michishita-cho, Nakamura-ku, Nagoya 453-8511, Japan; 2 Department of Surgical Pathology, School of Medicine, Aichi Medical University, 1-1 Yazakokarimata, Nagakute 480-1195, Japan; 3 Division of Urology, Harasanshin Hospital, Fukuoka 812-0033, Japan; yokoa@harasanshin.or.jp; 4 Department of Urology, Jikei University School of Medicine, Tokyo 105-8461, Japan; tkimura@jikei.ac.jp (T.K.); 0529@jikei.ac.jp (K.M.); 5 Japan Clinical Oncology Group Data Centre/Operations Office, National Cancer Centre Hospital, Tokyo 104-0045, Japan; keisasak@ncc.go.jp; 6 Department of Urology, Graduate School of Medical Sciences, Kyushu University, Fukuoka 812-8582, Japan; shiota.masaki.101@m.kyushu-u.ac.jp (M.S.); eto.masatoshi.717@m.kyushu-u.ac.jp (M.E.); 7 Department of Urology, Faculty of Medicine, Kagawa University, Miki-chō 761-0793, Japan; kato.takuma@kagawa-u.ac.jp (T.K.); sugimoto.mikio@kagawa-u.ac.jp (M.S.); 8 Department of Urology, Faculty of Medicine, University of Tsukuba, Tsukuba 305-8577, Japan; tkawahar@md.tsukuba.ac.jp (T.K.); nishiuro@md.tsukuba.ac.jp (H.N.); 9 Department of Urology, Kitazato University School of Medicine, Sagamihara 252-0374, Japan; koguchi.dai@kitasato-u.ac.jp; 10 Department of Urology, Shikoku Cancer Center, Matsuyama 791-0280, Japan; hashine.katsuyoshi.vc@mail.hosp.go.jp; 11 Department of Urology, Faculty of Medicine, University of Toyama, Toyama 930-0194, Japan; hkitamur@med.u-toyama.ac.jp

**Keywords:** prostate cancer, prostatectomy, salvage radiotherapy (SRT), salvage hormonal therapy (SHT), pathological predictive factor, intraductal carcinoma of the prostate (IDC-P), Gleason pattern 5

## Abstract

Background/Objectives: The multicenter randomized trial JCOG0401 showed that initial salvage radiotherapy (SRT) before salvage hormonal therapy (SHT) significantly prolonged time to treatment failure (TTF) compared with SHT alone. This central pathology analysis aimed to explore pathological features potentially associated with reduced relative benefit from SRT, while distinguishing prognostic from predictive effects. Methods: We re-analyzed 167 patients from JCOG0401 (SHT: 81, SRT ± SHT: 86). Prostatectomy specimens were re-evaluated, focusing on tertiary Gleason pattern (GP) 5 and intraductal carcinoma of the prostate (IDC-P). Cox proportional hazards models assessed exploratory interaction effects between pathological features and the treatment effect of bicalutamide on TTF. Pathological subgroups in which SRT failed to improve TTF over SHT alone were defined as having reduced benefit from SRT. Results: Adverse pathological features associated with shorter TTF with limited SRT included Gleason score (≥8), GP5, tertiary GP5, IDC-P, positive surgical margins, lymphovascular invasion, and advanced pathological T stage. Exploratory Interaction analyses suggested that IDC-P and tertiary GP5 may be associated with reduced relative benefit from SRT. Among patients without IDC-P, SRT significantly improved TTF (HR 0.330, 95%CI 0.161–0.673), whereas no significant benefit was observed in those with IDC-P (HR: 0.771, 95% CI: 0.446–1.332). Similarly, the absence of tertiary GP5 was associated with a marked benefit from SRT (HR: 0.099, 95% CI: 0.021–0.466), whereas this effect was not observed with tertiary GP5 (HR: 0.760, 95% CI: 0.400–1.443). Conclusions: IDC-P and tertiary GP5 may represent pathological features associated with reduced relative benefit from SRT after radical prostatectomy. These findings are exploratory and hypothesis-generating and require prospective validation in independent cohorts. Careful pathological evaluation may help identify patients who could benefit from treatment intensification or alternative postoperative strategies.

## Simple Summary

After radical prostatectomy, some patients experience biochemical recurrence, and salvage radiotherapy is commonly used to delay disease progression; however, its benefit is not uniform, and reliable predictors of response are lacking. This exploratory post hoc analysis re-evaluated pathological specimens from a multicenter randomized clinical trial to investigate pathological features potentially associated with reduced relative benefit from radiotherapy. Detailed pathological assessment focused on adverse morphological patterns, including intraductal carcinoma of the prostate and tertiary high-grade tumor components. These features were associated with poorer outcomes overall, suggesting prognostic relevance, and exploratory interaction analyses further suggested that they may be associated with reduced relative benefit from salvage radiotherapy. In contrast, patients without these features appeared to derive greater benefit from radiotherapy. These findings are hypothesis-generating and highlight the potential value of contemporary pathological evaluation for postoperative risk stratification and treatment personalization. Further prospective validation incorporating modern imaging and molecular markers is warranted.

## 1. Introduction

After radical prostatectomy for prostate cancer, both adjuvant radiotherapy and salvage radiotherapy (SRT) are available options for managing biochemical recurrence. However, the results from several randomized controlled trials have not demonstrated a clear benefit of adjuvant radiotherapy, and SRT is now considered the standard approach [1,2,3]. In addition to radiotherapy, hormonal therapy is also used as a salvage treatment for biochemical recurrence. Nevertheless, the precise indications and optimal sequencing of these therapies remain uncertain, and multiple studies have been conducted to address this issue.

The JCOG0401 trial, a multicenter, randomized, open-label, phase III study, enrolled patients with PSA recurrence (0.4–1.0 ng/mL) after radical prostatectomy and randomized them to salvage hormonal therapy (SHT) alone or SRT to the prostate bed alone (64.8 Gy) followed by the same hormonal treatment strategy in cases of treatment failure. The study demonstrated that patients with biochemical recurrence after radical prostatectomy derived greater benefit from SRT administered before salvage hormonal therapy than from SHT alone, with a median follow-up duration of 5.5 years [4]. Based on these clinical data, a nomogram was also developed to predict time to treatment failure (TTF) following SRT [5]. Notably, 31% of patients who received initial SRT did not require subsequent SHT because no treatment failure occurred. However, as not all patients benefit from salvage therapy, further clarification of patient selection criteria is essential.

The role of pathological factors in predicting outcomes following salvage therapies, such as SRT and SHT, remains insufficiently defined. Commonly evaluated factors include pT stage, Gleason score, surgical margin status, and lymph node metastasis [6,7,8]. Since the initiation of JCOG0401 in 2005, a major pathological criterion for prostate cancer, the Gleason score, has been revised. For example, all cribriform patterns are now classified as Gleason pattern 4 with aggressive features [9]. Additional concepts, such as tertiary Gleason pattern 5 (tGP5) and intraductal carcinoma of the prostate (IDC-P), have also been recognized as markers of aggressive disease, reflecting evolving pathological understanding. More recently, the European Association of Urology (EAU) guidelines have incorporated pathological features into risk stratification for biochemical recurrence after prostatectomy, with factors such as Gleason score ≥ 8 classified as high risk [10]. These updated criteria were not considered in the original analysis of JCOG0401, as they were not available at that time.

This central pathology review analysis of JCOG0401, based on the latest concept, aimed to identify currently used pathological predictors of therapeutic response, with a particular focus on patient profiles unlikely to benefit from the addition of radiotherapy. Given the time elapsed since the trial’s initiation, prostatectomy specimens were reviewed.

## 2. Materials and Methods

### 2.1. Trial Design

The JCOG0401 trial evaluated the efficacy of SRT combined with SHT versus SHT alone in patients with PSA failure after radical prostatectomy [4]. Eligible patients had localized prostate cancer (cT1–2N0M0) with rising PSA levels (0.4–1.0 ng/mL) but no clinical evidence of recurrence. Participants were randomly assigned (1:1) using a minimization method. Patients in the SHT arm received bicalutamide (80 mg/day), with a switch to LH-RH analogs upon treatment failure. Those in the SRT ± SHT arm received external beam radiotherapy (64.8 Gy) and, subsequently, SHT if treatment failure occurred. The primary endpoint was TTF on bicalutamide. The trial demonstrated that initial SRT significantly prolonged TTF compared with SHT alone.

For this ancillary study, new unstained slides were prepared by cutting sections from preserved radical prostatectomy tissue blocks, followed by Hematoxylin and Eosin staining and immunostaining for PTEN and ERG. Monoclonal antibodies against ERG (EP111, dilution 1:100) and PTEN (SP218, dilution 1:100) were used for immunohistochemistry. ERG expression was considered positive when a cluster of tumor nuclei demonstrated positive staining. PTEN expression was considered “PTEN loss” when at least 90% of tumor cells showed absence of staining. “Not applicable” refers to cases in which Gleason pattern 5 was already included as the primary or secondary Gleason pattern, such as GS 3 + 5, 5 + 3, 4 + 5, 5 + 4, or 5 + 5; therefore, tertiary Gleason pattern 5 could not be assigned. All slides were reviewed by a single genitourinary pathologist (T.T.) according to the International Society of Urological Pathology (ISUP) 2019 criteria [11]. Forty-three patients were excluded because their institutions declined to participate in the re-analysis or because resected specimens were unavailable due to age-related degradation or inadequate preservation.

### 2.2. Statistical Analysis

Subgroup analyses for individual pathological factors were performed using the Kaplan-Meier method, with differences between treatment arms assessed using the log-rank test. Cox proportional hazards regression models were applied to estimate hazard ratios (HRs) and 95% confidence intervals (CIs). Interaction *p*-values between treatment and covariates were calculated to identify predictive factors, with *p* < 0.15 considered indicative of interaction. Other statistical analyses were considered significant at *p* < 0.05. Pathological subgroups in which SRT ± SHT failed to improve TTF compared with SHT alone were defined as treatment-resistant factors.

Second, univariable and multivariable Cox proportional hazards regression analyses were conducted for TTF on SHT within each treatment arm (SRT ± SHT and SHT alone) to identify risk factors for treatment failure. Because the number of candidate covariates was relatively large compared with the number of observed events, inclusion of all variables in the multivariable Cox model was considered likely to cause overfitting and unstable HR estimates. Therefore, a step-wise variable selection procedure was used with significance levels of 0.15 for inclusion and 0.20 for exclusion. Potential predictors included pathological Gleason score on radical prostatectomy specimens (<8 vs. ≥8), Gleason pattern 5 (absent vs. present), tGP5 (absent vs. present), IDC-P (absent vs. present), surgical margin status (negative vs. positive), lymphovascular invasion (absent vs. present), pathological T stage (≤pT2 vs. ≥pT3), age (<65 vs. ≥65 years), PTEN expression (retained vs. loss), and ERG expression (negative vs. positive). Variables identified in each arm’s multivariable analysis and demonstrating a consistent treatment effect regardless of their presence were defined as risk factors. No adjustment for multiplicity was performed; therefore, the findings from these analyses should be considered hypothesis-generating and interpreted with caution.

All statistical analyses were conducted using SAS software, version 9.4 (SAS Institute, Cary, NC, USA).

### 2.3. Ethics Statement

This ancillary study was conducted in accordance with the Ethical Guidelines for Clinical Research and the Ethical Guidelines for Medical and Health Research Involving Human Subjects. The JCOG0401 trial was prospectively registered with the University Hospital Medical Information Network Clinical Trials Registry (identifier: C000000026). The study protocol for this ancillary analysis was approved by the institutional review board at each participating center prior to initiation.

## 3. Results

### 3.1. Patient Characteristics

Of the 210 patients enrolled in the JCOG0401 trial, 167 were included in this analysis (SHT: 81; SRT ± SHT: 86). Baseline patient characteristics are summarized in Table 1. The median age was 71 years (range, 53–79) in the SHT arm and 71.5 years (range, 54–79) in the SRT ± SHT arm. Performance status 1 was observed only in the SHT arm (*n* = 3). Overall, 48 patients (28.7%) were classified as ISUP Grade Group 5, and IDC-P was present in 69 patients (41.3%). Extraprostatic extension was identified in 38 patients in the SHT arm and 54 in the SRT ± SHT arm. Positive surgical margins were reported in 27 patients in the SHT arm and 35 in the SRT ± SHT arm. PTEN loss was detected in 12 patients in the SHT arm and 11 in the SRT ± SHT arm, whereas ERG positivity was identified in seven and two patients, respectively (Table 1).

### 3.2. Impact of SRT ± SHT on TTF of Bicalutamide: Subgroup Analysis by Pathological Factors

The log-rank test demonstrated differences in TTF on bicalutamide between the SHT and SRT ± SHT arms across several pathological factors. Predictive indicators of reduced relative benefit from SRT included Gleason score ≥ 8, presence of GP5, presence of tGP5, IDC-P positivity, negative surgical margin status, lymphovascular invasion, pathological stage T3, younger age, and ERG positivity (Table 2).

**Figure 1:**
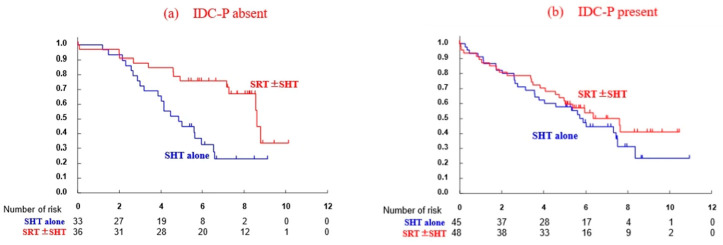

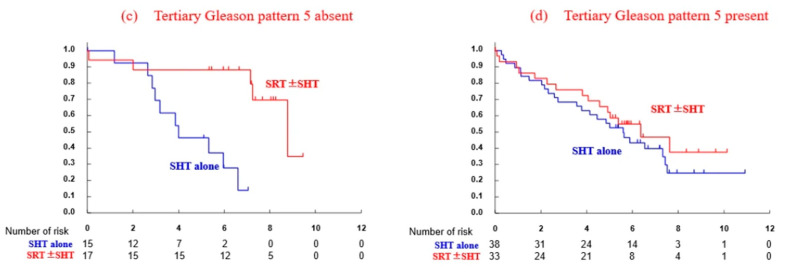
Kaplan–Meier estimates of relapse-free survival comparing treatment outcomes in the SHT arm and the SRT ± SHT arm. (**a**) IDC-P-negative patients; (**b**) IDC-P-positive patients; (**c**) Tertiary Gleason pattern 5-negative patients; (**d**) Tertiary Gleason pattern 5-positive patients. SHT = salvage hormone therapy; SRT = salvage radiation therapy.

**Figure 2:**
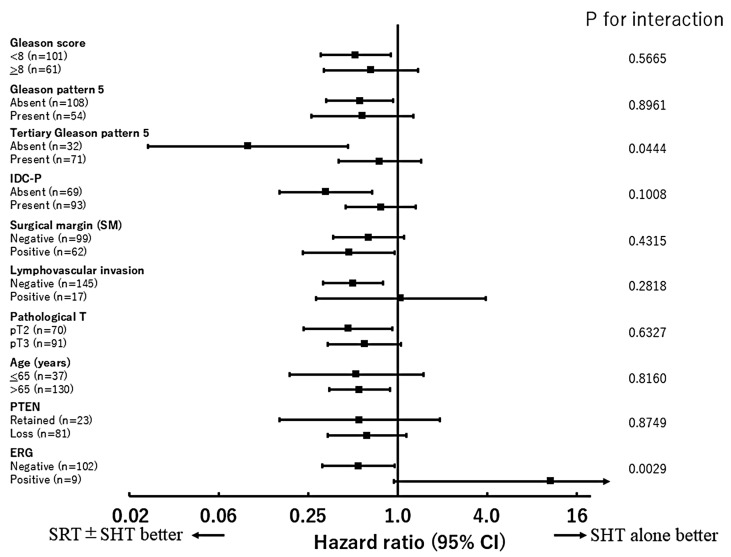
Forest plot of subgroup analyses.

In IDC-P-absent patients, the HR for disease progression in the SRT ± SHT arm was 0.330 (95% CI: 0.161–0.673, *p* = 0.0023), whereas in IDC-P-present patients the effect was attenuated (HR: 0.771, 95% CI: 0.446–1.332, *p* = 0.351) (Figure 1a,b), with evidence of interaction (*p* for interaction = 0.1008). Similarly, in tGP5-absent patients, the HR was 0.099 (95% CI: 0.021–0.466, *p* = 0.0034), whereas in tGP5-present patients the effect was attenuated (HR: 0.760, 95% CI: 0.400–1.443, *p* = 0.401) (Figure 1c,d), with a significant interaction (*p* for interaction = 0.044) (Figure 2). In ERG-negative patients, the hazard ratio (HR) was 0.548 (95% CI: 0.312–0.962, *p* = 0.0362), whereas in ERG-positive patients the effect was markedly attenuated (HR: 10.7, 95% CI: 0.951–120.8, *p* = 0.055), with a significant interaction (*p* for interaction = 0.0029). However, given the limited number of ERG-positive cases, these findings should be interpreted with caution, and their statistical robustness remains uncertain.

Univariable and multivariable Cox proportional hazards regression analyses were performed to assess potential risk factors associated with TTF in patients treated with bicalutamide. Despite a comprehensive evaluation of clinical and pathological variables, no significant predictors were identified in either the SHT or SRT ± SHT arms, both of which showed similar trends (Appendix A Table A1).

The results of the sensitivity analyses were generally consistent with the primary analysis, and no material changes in the overall conclusions were observed across alter-native model specifications.

## 4. Discussion

This ancillary exploratory analysis of JCOG0401 investigated pathological features potentially associated with reduced relative benefit from radiotherapy combined with hormonal therapy for postoperative recurrence of prostate cancer. IDC-P and tGP5 were associated with less apparent relative benefit from SRT in exploratory interaction analyses, although these findings should be interpreted cautiously given the retrospective post hoc nature of the study. Several high-risk features have been proposed for patients with biochemical recurrence following radical prostatectomy [6,7,8]. Established prognostic factors include pathological T stage, Gleason score, surgical margin status, and nodal involvement, all of which were evaluated in the JCOG0401 trial. However, the classification and diagnostic criteria for these factors have evolved through the ISUP 2014 and ISUP 2018 consensus meetings [12,13]. Accordingly, re-evaluating the potential prognostic and predictive relevance of these pathological features is of clinical interest.

IDC-P has been reported to be strongly associated with high-grade, high-volume invasive prostate cancer and with unfavorable clinical outcomes following radical prostatectomy [14,15]. Histologically, it presents as well-circumscribed lesions with intact basal cell layers surrounding distended ducts infiltrated by malignant epithelium [12,13]. A recent systematic review of localized prostate cancer treated with curative intent, encompassing 46 studies, reported that cases with cribriform pattern, intraductal carcinoma, or ductal adenocarcinoma had higher rates of biochemical recurrence, metastasis, and cancer-specific survival than conventional prostate cancer [16].

Several studies have reported that IDC-P-positive cases exhibit resistance to radiotherapy. Kwast et al. [17] first demonstrated, in an intermediate-risk cohort, that IDC-P strongly predicted early biochemical relapse. Similarly, Tom et al. [18] reported that IDC-P with a cribriform pattern was significantly associated with worse biochemical recurrence-free survival (HR: 4.22, *p* < 0.0001), distant metastasis-free survival (HR: 4.18, *p* = 0.01), and disease-specific survival (HR: 14.26, *p* = 0.0016) in a cohort of 237 patients treated with dose-escalated radiotherapy. Aizawa et al. [19] further showed that in high-risk prostate cancer patients treated with Intensity-Modulated Radiotherapy (IMRT) (median 78 Gy) plus hormonal therapy, IDC-P was associated with higher rates of biochemical failure (*p* = 0.04), clinical failure (*p* = 0.0031), and castration-resistant progression (*p* = 0.012) over a median follow-up of 8.4 years. However, these prior studies primarily demonstrated adverse prognostic associations rather than definitive predictive effects regarding radiotherapy benefit. Notably, all existing data on IDC-P in the context of radiotherapy are based on pathological diagnoses from biopsy specimens, and no comprehensive studies have examined IDC-P in SRT, where radical prostatectomy specimens are available. Because prostatectomy specimens generally allow higher detection rates of IDC-P compared with biopsy samples, due to reduced sampling error, the present study provides important added value [20].

The concept of tGP5 in prostate cancer pathology was introduced to account for the presence of a small component of high-grade (Gleason 5) tumor within an otherwise lower-grade cancer (GG2 or GG3), as incorporated into the 2005 and 2014 ISUP Gleason grading modifications [9,12]. Its predictive utility for PSA failure has been previously reported [21]. In the present study, tGP5 demonstrated differences between the two arms with respect to the additional benefit of radiotherapy, with a significant interaction *p*-value observed for tGP5, which was present in a lower proportion of cases. For ERG analysis, the number of evaluable cases was limited; therefore, these findings should be considered underpowered and exploratory. Thus, the findings should be interpreted with caution. PTEN and ERG expression have both been reported to be associated with IDC-P in a Western patient cohort [22]. However, the prevalence of these alterations is generally considered to be low in the Japanese population [23,24]. In the present cohort, the numbers of patients with PTEN loss and ERG positivity were small, limiting statistical power; therefore, further studies with larger sample sizes are warranted to validate these findings in Japanese patients.

The EAU guidelines identify a Gleason score of ≥8 in radical prostatectomy specimens as a high-risk factor for postoperative recurrence [8]. A recent study proposed a prognostic risk scoring system for SRT in post-prostatectomy patients that incorporates Gleason score, PSA at the start of SRT, and margin status [25]. However, in large-scale trials, detailed microscopic examination by a central pathologist—such as that performed in the present study—is not feasible [26]. Consequently, comparable analyses cannot be conducted in those settings, underscoring the significance of our study.

IDC-P and tGP5, identified in this study as pathological features potentially associated with reduced relative benefit from SRT, should not be interpreted as definitive predictive biomarkers. Prior to the analysis, we anticipated that patients with these features would benefit from radiation therapy. However, as shown in Figure 1 and Table 2, radiotherapy improved prognosis only in patients without these risk factors, whereas those with them derived no significant benefit—contrary to expectations. This unexpected finding is particularly noteworthy. These unexpected findings are hypothesis-generating and warrant validation in larger independent cohorts.

The absence of statistically significant factors in the univariable and multivariable Cox regression analyses may partly be explained by methodological differences relative to prior studies. While conventional analyses often define the index date as the time of surgery, this study measured TTF from the randomization date after PSA recurrence. This difference in the starting point of follow-up may have influenced event timing and limited the ability to detect statistically significant prognostic factors.

Rather than excluding such patients from SRT, these findings may help identify patients who could benefit from treatment intensification or alternative multimodal strategies. One potential strategy, demonstrated in the EMBARK trial, is systemic therapy with enzalutamide, an androgen receptor pathway inhibitor (ARPI), combined with an LHRH analog for patients with high-risk biochemical recurrence of prostate cancer [27]. The PRESTO trial evaluating apalutamide in high-risk biochemically relapsed castration-sensitive prostate cancer has been reported [28]. In addition, several reports have suggested the efficacy of docetaxel or ARPIs in the treatment of IDC-P [29,30]; however, a consensus has not yet been reached, and the development of novel therapeutic approaches remains an important need. Future studies incorporating prospective validation, molecular biomarkers, and modern imaging modalities such as PSMA PET will be important to clarify the clinical utility of these pathological features in treatment stratification.

Importantly, the present study has several limitations. First, this was a retrospective post hoc analysis with a relatively limited sample size and multiple subgroup comparisons, increasing the risk of false-positive findings. Second, several subgroup analyses included small patient numbers. In addition, a substantial proportion of patients had “Not Done” status for N stage, PTEN, and ERG assessments, which may have further reduced statistical power and introduced potential bias. Third, selection bias may have been introduced because not all patients enrolled in the original JCOG0401 trial were included in this pathological analysis. Fourth, modern imaging modalities such as PSMA PET were not available during the study period, which may limit applicability to current clinical practice. Finally, treatment approaches for biochemical recurrence have evolved substantially since the accrual period of JCOG0401, including broader use of LHRH-based androgen deprivation therapy, androgen receptor pathway inhibitors, and modern imaging-guided management. Accordingly, caution is required when extrapolating the present results directly to contemporary clinical practice.

## 5. Conclusions

In this exploratory ancillary analysis of JCOG0401, IDC-P and tGP5 were identified as pathological features potentially associated with reduced relative benefit from SRT combined with SHT for biochemical recurrence after radical prostatectomy. These findings should be interpreted as hypothesis-generating rather than definitive evidence of predictive biomarkers. While these pathological features were also associated with adverse outcomes overall, exploratory interaction analyses suggested that they may influence the relative benefit derived from SRT. Rather than excluding patients from SRT, these observations may help identify patients who could benefit from treatment intensification or alternative multimodal strategies. The findings highlight the value of detailed pathological assessment of radical prostatectomy specimens for enabling more precise patient stratification. Further prospective studies incorporating larger cohorts, molecular biomarkers, and modern imaging modalities such as PSMA PET are warranted to validate these observations and clarify their clinical utility in postoperative treatment decision-making.

## Figures and Tables

**Table 1:** Clinical and pathological backgrounds.

	SHT Arm	SRT ± SHT Arm	Total
	*n* = 81	*n* = 86	*n* = 167
Age median (min–max)	71 (53–79)	71.5 (54–79)	
Performance status			
0	78	86	164
1	3	0	3
Gleason score			
3 + 3	0	2	2
3 + 4	29	23	52
3 + 5	2	3	5
4 + 3	23	24	47
4 + 4	2	5	7
4 + 5	18	20	38
5 + 3	0	1	1
5 + 4	3	5	8
5 + 5	1	1	2
not done	3	2	5
ISUP Grade Group			
GG1	0	2	2
GG2	29	23	52
GG3	23	24	47
GG4	4	9	13
GG5	22	26	48
not done	3	2	5
Tertiary Gleason pattern 5			
absent	15	17	32
present	38	33	71
not applicable	20	31	51
not done	5	2	7
unknown	3	3	6
IDC-P			
absent	45	48	93
present	33	36	69
not done	3	2	5
Pathological T stage			
2	40	30	70
3a	30	47	77
3b	7	7	14
not done	3	2	5
unknown	1	0	1
Extraprostatic extension			
negative	40	29	69
positive	38	54	92
not applicable	0	1	1
not done	3	2	5
Lymphovascular invasion			
negative	70	75	145
positive	8	9	17
not done	3	2	5
Seminal vesicle invasion			
negative	70	76	146
positive	7	7	14
not done	4	3	7
Surgical margin			
negative	50	49	99
positive	27	35	62
not applicable	1	0	1
not done	3	2	5
Pathological N stage			
N0	53	58	111
N1	0	0	0
not applicable	0	1	1
not done	27	26	53
unknown	1	1	2
PTEN			
loss	12	11	23
retained	38	44	82
not applicable	4	2	6
not done	26	28	54
unknown	1	1	2
ERG			
negative	47	55	102
positive	7	2	9
not done	26	28	54
unknown	1	1	2

GG = ISUP Grade Group; SHT = salvage hormone therapy; SRT = salvage radiation therapy.

**Table 2:** Subgroup analyses.

Variables	Category	Arm	Event	HR (95% CI)	*p*-Value
Gleason score	<8	SHT	35/52	1	
		SRT ± SHT	21/49	0.524 (0.304–0.904)	0.0201
	≥8	SHT	14/26	1	
		SRT ± SHT	16/35	0.663 (0.320–1.375)	0.2699
Gleason pattern 5	Absent	SHT	36/54	1	
		SRT ± SHT	24/54	0.558 (0.332–0.939)	0.028
	Present	SHT	13/24	1	
		SRT ± SHT	13/30	0.581 (0.265–1.274)	0.1755
tGP5	Absent	SHT	10/15	1	
		SRT ± SHT	5/17	0.099 (0.021–0.466)	0.0034
	Present	SHT	25/38	1	
		SRT ± SHT	15/33	0.76 (0.400–1.443)	0.4013
IDC-P	Absent	SHT	21/33	1	
		SRT ± SHT	13/36	0.33 (0.161–0.673)	0.0023
	Present	SHT	28/45	1	
		SRT ± SHT	24/48	0.771 (0.446–1.332)	0.3507
Surgical margin	Negative	SHT	31/50	1	
		SRT ± SHT	22/49	0.641 (0.370–1.108)	0.1113
	Positive	SHT	17/27	1	
		SRT ± SHT	15/35	0.471 (0.232–0.957)	0.0374
Lymphovascular invasion	Negative	SHT	45/70	1	
		SRT ± SHT	32/75	0.502 (0.317–0.797)	0.0034
	Positive	SHT	4/8	1	
		SRT ± SHT	5/9	1.051 (0.282–3.920)	0.9414
Pathological T stage	pT2	SHT	25/40	1	
		SRT ± SHT	13/30	0.465 (0.235–0.920)	0.0278
	pT3	SHT	24/37	1	
		SRT ± SHT	24/54	0.599 (0.338–1.061)	0.0787
Age	≤65	SHT	13/21	1	
		SRT ± SHT	5/16	0.529 (0.188–1.490)	0.2279
	>65	SHT	38/60	1	
		SRT ± SHT	32/70	0.556 (0.346–0.892)	0.015
PTEN	Loss	SHT	7/12	1	
		SRT ± SHT	4/11	0.555 (0.160–1.926)	0.3541
	Retained	SHT	23/38	1	
		SRT ± SHT	20/44	0.626 (0.341–1.149)	0.1308
ERG	Negative	SHT	28/47	1	
		SRT ± SHT	23/55	0.548 (0.312–0.962)	0.0362
	Positive	SHT	5/7	1	
		SRT ± SHT	2/2	10.719 (0.951–120.841)	0.055

CI = confidence interval; SHT = salvage hormone therapy; SRT = salvage radiation therapy; tGP5 = tertiary Gleason pattern 5; HR = Hazard ratio.

## Data Availability

The data presented in this study are available on request from the corresponding author due to privacy.

## Abbreviations

The following abbreviations are used in this manuscript:

ARPI	Androgen receptor pathway inhibitor
CI	Confidence interval
EAU	The European Association of Urology
IDC-P	Intraductal carcinoma of the prostate
LHRH	Luteinizing hormone-releasing hormone
HR	Hazard ratio
ISUP	International Society of Urological Pathology
SHT	Salvage hormone therapy
SRT	Salvage radiation therapy
tGP5	Tertiary Gleason pattern 5
TTF	Time to treatment failure

## Appendix A

**Table A1:** Univariate and multivariate Cox proportional hazards regression analysis for prediction of time to treatment failure of bicaltamide.

	SHT: *n* = 81					SRT ± SHT: *n* = 86			
Variables	Category	Univariable Analysis		Multivariable Analysis		Univariable Analysis		Multivariable Analysis	
		HR (95% CI)	*p*-Value	HR (95% CI)	*p*-Value	HR (95% CI)	*p*-Value	HR	*p*-Value
		(95% CI)		(95% CI)		(95% CI)		(95% CI)	
Gleason score	<8	1				1			
	≥8	0.679	0.2225			0.899	0.7489		
		(0.365–1.264)				(0.468–1.726)			
Gleason pattern 5	Absent	1				1			
	Present	0.689	0.2519			0.77	0.4502		
		(0.365–1.303)				(0.390–1.518)			
Tertiary Gleason pattern 5	Absent	1				1			
	Present	0.717	0.385			2.467	0.0851		
		(0.338–1.520)				(0.883–6.895)			
IDC-P	Absent	1		1		1		1	
	Present	0.804	0.4531	0.365	0.0324	1.634	0.156	12.76	0.0274
		(0.455–1.421)		(0.137–0.973)		(0.829–3.222)		(1.329–122.510)	
Surgical margin	Negative	1				1			
	Positive	1.199	0.548			0.857	0.6472		
		(0.663–2.168)				(0.442–1.660)			
Lymphovascular invasion	Negative	1		1		1		1	
	Positive	0.744	0.5742	6.375	0.0448	1.611	0.3264	8.095	0.242
		(0.266–2.084)		(1.192–34.095)		(0.622–4.174)		(1.313–49.898)	
Pathological T stage	pT2	1				1			
	pT3	1.068	0.8173			1.315	0.4283		
		(0.610–1.871)				(0.668–2.587)			
Age	≤65	1				1		1	
	>65	1.141	0.6811			1.333	0.5515	5.494	0.0919
		(0.607–2.145)				(0.518–3.428)		(0.758–39.842)	
PTEN	Retained	1		1		1			
	Loss	1.126	0.7835	2.329	0.1291	1.281	0.6551		
		(0.482–2.630)		(0.759–7.145)		(0.432–3.794)			
ERG	Negative	1				1		1	
	Positive	0.784	0.6221			9.546	0.0046	413.537	0.0007
		(0.297–2.067)				(2.005–45.449)		(12.713–13,451.87)	

SHT = salvage hormone therapy; SRT = salvage radiation therapy.
